# Childhood interstitial lung diseases in immunocompetent children in Australia and New Zealand: a decade’s experience

**DOI:** 10.1186/s13023-017-0637-x

**Published:** 2017-07-25

**Authors:** Vishal Saddi, Sean Beggs, Bruce Bennetts, Joanne Harrison, Neil Hime, Nitin Kapur, Jill Lipsett, Lawrence M. Nogee, Amy Phu, Sadasivam Suresh, André Schultz, Hiran Selvadurai, Stephanie Sherrard, Roxanne Strachan, Julian Vyas, Yvonne Zurynski, Adam Jaffé

**Affiliations:** 1 Department of Respiratory Medicine, Discipline of Paediatrics, Sydney Children’s Hospital, Randwick, Sydney, NSW 2031 Australia; 2 Department of Paediatrics, Royal Hobart Hospital, Hobart, TAS 7000 Australia; 3 Department of Molecular Genetics, The Children’s Hospital at Westmead, Sydney, NSW 2145 Australia; 4 Discipline of Paediatrics and Child Health, Sydney Medical School, The University of Sydney, Sydney, Australia; 5 Department of Respiratory Medicine, The Children’s Hospital, Melbourne, VIC 3052 Australia; 6 Australian Paediatric Surveillance Unit, Kids Research Institute, Sydney, NSW 2145 Australia; 7 Department of Respiratory Medicine, Lady Cilento Children’s Hospital, Brisbane, QLD 4101 Australia; 8 Anatomical Pathology, S.A. Pathology, Women’s and Children’s Hospital, Adelaide, South Australia 5154 Australia; 9 Eudowood Neonatal Pulmonary Division, Department of Pediatrics, Johns Hopkins University School of Medicine, Baltimore, MD 21287 USA; 10 Department of Respiratory Medicine, Princess Margaret Hospital for Children, Perth, WA 6008 Australia; 11 Department of Respiratory Medicine, The Children’s Hospital at Westmead, Sydney, NSW 2145 Australia; 12 Department of Respiratory Paediatrics, Starship Children’s Hospital, Auckland, 1023 New Zealand; 13 School of Women’s and Children’s Health, Faculty of Medicine, University of New South Wales, Sydney, NSW 2031 Australia

**Keywords:** Interstitial lung disease, chILD syndrome, Australia, New Zealand

## Abstract

**Background:**

Childhood interstitial lung disease (chILD) represents a rare heterogeneous group of respiratory disorders. In the absence of randomized controlled clinical trials, global collaborations have utilized case series with an aim to standardising approaches to diagnosis and management. Australasian data are lacking. The aim of this study was to calculate prevalence and report the experience of chILD in Australasia over a decade.

**Methods:**

Paediatric pulmonologists in Australia and New Zealand involved in the care of patients aged 0–18 years with chILD completed a questionnaire on demographics, clinical features and outcomes, over a 10 year period. These data, together with data from the 2 reference genetics laboratories, were used to calculate prevalence.

**Results:**

One hundred fifteen cases were identified equating to a period prevalence (range) of 1.5 (0.8–2.1) cases/million for children aged 0–18years. Clinical data were provided on 106 patients: the <2 year group comprised 66 children, median age (range) 0.50 years (0.01–1.92); the ≥2 year group comprised 40 children, median age 8.2 years (2.0–18.0). Management approach was heterogeneous. Overall, 79% of patients had a good clinical outcome. Mortality rate was 7% in the study population.

**Conclusion:**

chILD is rare in Australasia. This study demonstrates variation in the investigations and management of chILD cases across Australasia, however the general outcome is favorable. Further international collaboration will help finesse the understanding of these disorders.

## Background

Childhood interstitial lung disease (chILD) is a heterogenous group of rare chronic respiratory disorders in children, most prevalent in early infancy. It is associated with variable lung pathology which often impairs gas exchange [1]. chILD is characterized by dyspnoea, tachypnoea, crackles, hypoxaemia, failure to thrive and results in significant morbidity and mortality [2, 3]. The term interstitial lung disease (ILD) is perhaps a misnomer as the associated disease process may affect the alveoli, airways, blood vessels, lymphatic channels, and pleural spaces in addition to the interstitium [4]; hence the term “diffuse parenchymal lung disease” is often used [5].

The causes of chILD are multifactorial and include genetic, developmental, inflammatory and infectious determinants; in many cases the aetiology is unknown. The most common treatments are corticosteroids, hydroxychloroquine, azithromycin, nutritional support and oxygen therapy which may be required for many years. The classification of chILD has evolved as new disorders have been identified. As these conditions are very rare, there are limited scientific data on which to inform appropriate management, with a compelling need to develop this evidence base through national and international collaboration.

In Australia and New Zealand there has been growing interest in the systematic collection and provision of accurate data to develop effective policy, health and community services for rare diseases, including lung disease [6]. In contrast to other countries, research into chILD in Australia and New Zealand has been limited and confined to case reports and systematic reviews [1, 7]. Although Australia and New Zealand have highly developed health systems, their population characteristics bring unique challenges to managing patients with rare diseases. Their overall populations are relatively small and widely dispersed with a low population density. Small numbers of chILD patients in any one location in Australasia emphasise the need for collaboration between treating hospitals to consolidate the knowledge and increase awareness of chILD in the Australasian population.

The aims of this study were to calculate the prevalence and describe the demographics, clinical features and outcomes of chILD in Australia and New Zealand.

## Methods

We conducted a retrospective review of children younger than 18 years of age at diagnosis who were evaluated for ILD in the tertiary paediatric hospitals in Australia and New Zealand during the period January 2003 to December 2013. A questionnaire was developed based on a survey of idiopathic interstitial pneumonitis in the United Kingdom [8]. The questionnaire included information on patient demographics, clinical symptoms at first hospital presentation, family history, risk factors, investigations, treatments and clinical outcomes.

All eleven tertiary paediatric hospitals in Australia, and a tertiary paediatric hospital in Auckland, New Zealand were invited to participate in the survey. A paediatric respiratory physician from each centre coordinated the data collection of all children treated for chILD at their respective hospital. Patients were identified from review of hospital databases and by physician recall. A questionnaire was completed following extraction of relevant information from clinical records and data were entered into an Excel database.

Children (0–18 years) were included in the study if they were immunocompetent, either had a confirmed diagnosis of chILD or, in those in whom diagnosis was suspected, satisfied the 2004 European taskforce on chILD’s definition: the presence of persisting of respiratory symptoms, and/or diffuse infiltrates on CT scanning or abnormal pulmonary function tests with evidence of a restrictive ventilatory defect, and persistence of these findings for greater than 3 months [9].

In keeping with the North American classification system, the data were stratified by patients aged less than 2 years and aged 2–18 years [5]. Results were presented as descriptive data with frequency quantified as numbers and percentages of the total reported cases.

To improve the accuracy of the prevalence calculation, data on potential further cases of surfactant protein deficiencies were obtained from the two genetics laboratories (Johns Hopkins University Hospital, Baltimore, USA and The Children’s Hospital at Westmead, Sydney, Australia) that had performed investigations for surfactant deficiencies in Australasian paediatric patients during the study period. These laboratories supplied minimal demographic data (initials and date of birth) to allow identification of potential duplicates from the reporting physicians, together with details of genetic tests and diagnosis; no detailed clinical information was requested. Prevalence was calculated from the number of cases from 1st January 2003 to 31st December 2013. The denominator used to calculate population prevalence was the age specific population estimates from the Australian Bureau of Statistics and New Zealand Statistics Bureau for mid-year 2008 [10, 11].

Human research ethics approval was obtained from all centers involved in this study. Informed consent for genetic testing was obtained for all subjects investigated under a research protocol.

## Results

Eight tertiary hospitals across all States in Australia, and a tertiary paediatric hospital in Auckland, NZ participated in the study. A total of 108 cases of chILD were identified through the physician network; the majority (73%) by treating physician recall. One case with diagnostic data only was used in the estimate of prevalence but excluded from clinical data (107 patients). Of the eight participating hospitals in Australia: 32 cases of chILD were identified in Queensland from the Royal Children’s Hospital (*n* = 14) and Mater Hospital (*n* = 18); 18 cases in New South Wales from The Children’s Hospital at Westmead (*n* = 12), Sydney Children’s Hospital (*n* = 5), and John Hunter Hospital (*n* = 1); 16 cases from the Princess Margaret Hospital in Western Australia; 13 cases from The Royal Children’s Hospital in Victoria; 1 case from the Royal Hobart Hospital in Tasmania and 28 cases from The Starship Hospital in Auckland, NZ.

Between 2005 and 2011 blood from 16 children (2 from NZ) was sent for genetic studies to Johns Hopkins University, Baltimore, Maryland, USA. After excluding duplicates from those reported by the respiratory physicians, 4 additional cases of chILD were identified with the following diagnoses: *ABCA3* deficiency (*n* = 3); surfactant protein B deficiency (*n* = 1). Between 2011 and 2013, blood from 47 children (6 from NZ) was sent for genetic studies to The Children’s Hospital Westmead, Sydney, Australia. After excluding duplicates from those reported to us by the respiratory physicians, 3 additional cases of chILD were identified with the following diagnosis: *ABCA3* deficiency (*n* = 2) and surfactant protein C deficiency (*n* = 1). The period prevalence (range) of chILD in Australasia between 2003 and 2013 was 1.5 (0.8–2.1)/million for children aged 0–18 years.

The specific diagnoses are presented in Table 1.

**Table 1:** Frequency of reported chILD diagnoses (*n* = 115)

Undefined interstitial lung disease	21
Disorders More Common in Infancy	
NEHI	15
*ABCA3* deficiency	8
Lung hypoplasia (various causes)	6
Idiopathic bronchiolitis of infancy	4
Surfactant protein C deficiency	4
Nonspecific interstitial pneumonitis	4
Acinar/Alveolar capillary dysplasia	3
Surfactant Protein B deficiency	2
Chronic pneumonitis of infancy	2
Pulmonary interstitial glycogenosis	2
Interstitial lung disease with chronic pneumonitis of infancy and possible bronchiolitis obliterans	1
Diffuse pulmonary development disorder with patchy pulmonary interstitial glycogenosis plus bronchiolitis obliterans organizing pneumonia	1
Cellular interstitial pneumonitis of infancy	1
Disorders of Normal Host	
Pulmonary hemosiderosis	5
Obliterative bronchiolitis (various causes)	3
Follicular bronchiolitis	3
Pulmonary vasculitis/capillaritis	2
Eosinophilic pneumonia	1
ILD secondary to pertussis pneumonia	1
Interstitial pneumonitis post adenovirus bronchiolitis	1
Probable aspiration with respiratory syncytial virus infection	1
Post infectious interstitial lung disease	1
Idiopathic pulmonary fibrosis	1
Pulmonary fibrosis, giant cell pneumonitis on biopsy	1 1
Usual interstitial pneumonitis	
Desquamative interstitial pneumonitis	1
Lymphoid interstitial pneumonia	1
Nodular lymphoid hyperplasia	1
Disorders Related to Systemic Diseases	
ILD associated with juvenile dermatomyositis/scleroderma	4
ILD associated with Busulphan/Melphalan/Radiation toxicity	4
ILD with lymphangio-leiomyomatosis/Tuberous sclerosis	2
Anti-synthetase syndrome associated with non-specific interstitial pneumonia	1
ILD associated with mixed connective tissue disease	1
ILD associated with Erdheim Chester disease	1
ILD associated with Niemann Pick type B disease	1
ILD associated with systemic sclerosis	1
Pulmonary sarcoidosis	1
ILD associated with Langerhans cell histiocytosis	1

NEHI, Neuroendocrine hyperplasia of infancy; ABCA3, ATP-binding cassette sub-family A member 3 protein

More males (57%) were reported and in nearly 8% of all cases, siblings were affected by a similar disease, whilst 4% of cases had other affected family members; genetic associations were more common in children aged <2 years with an affected sibling in 12% compared to nearly 3% in the 2–18 years group (Table 2).

**Table 2:** Clinical characteristics of chILD patients at initial presentation to reporting centre

	*N* (%)	All cases	Diagnosis < 2 years		Diagnosis ≥ 2 years	
		No. reported (Max 107)	*N* (%)	No. reported (Max 66)^a^	*N* (%)	No. reported (Max 40)^a^
*Sex:* male/female	59/45 (57/43)	104	40/26 (61/39)	66	19/18 (51/49)	37
*Age at Diagnosis (years)*	0.01–18.00		0.01–1.92		2.00–18.00	
*Median (range)*			0.50		8.20	
*Ethnic Origin*		57		27		30
Caucasian	41 (72.0)		21 (77.8)		20 (66.8)	
Aboriginal	2 (3.5)		2 (7.4)		0	
Maori	6 (10.5)		2 (7.4)		4 (13.3)	
Pacific Islander	2 (3.5)		0		2 (6.6)	
Other	6 (10.5)		2 (7.4)		4 (13.3)	
*Parental Consanguinity*	3 (5.8)	51	0	26	3 (12.0)	25
*Family History*						
Affected siblings	7 (8.1)	86	6 (12.0)	50	1 (2.7)	36
Other family members affected	3 (4.0)	75	3 (7.5)	40	0	35
*Symptoms*						
Cough	51 (56.0)	91	24 (47.0)	51	26 (66.7)	39
Tachypnoea	76 (76.7)	99	54 (91.5)	59	22 (55.0)	40
Failure to thrive	35 (40.2)	87	26 (53.1)	49	9 (23.7)	38
Fever	12 (13.8)	87	7 (14.6)	48	5 (12.8)	39
Exertion dyspnoea	42 (56.0)	75	16 (41.0)	39	26 (72.2)	36
Dyspnoea at rest	43 (50.0)	86	26 (53.1)	49	17 (45.9)	37
*Physical Findings*						
Cyanosis	22 (22.7)	97	11 (19.3)	57	11 (28.2)	39
Clubbing	13 (14.3)	91	3 (5.9)	51	10 (25.6)	39
Crackles	56 (58.3)	96	29 (51.8)	56	26 (66.7)	39
Wheeze	20 (22.7)	88	12 (23.5)	51	8 (21.6)	37
*Birth History*						
*Mode of delivery*						
Spontaneous vaginal birth	61 (77.2)	79	40 (74.1)	54	20 (83.3)	24
*Gestation*						
< 37 weeks	18 (21.4)	84	15 (25.4)	59	3 (12.0)	25
≥ 37 weeks	66 (78.6)	84	44 (74.6)	59	22 (88.0)	25
*Respiratory distress at birth*	31 (41.3)	75	26 (57.8)	45	4 (13.8)	29
Intubation at birth	20 (24.7)	81	18 (38.3)	47	2 (6.1)	33
Surfactant at birth	7 (9.2)	76	7 (15.9)	44	0	32

^*a*^ *Age at diagnosis was not documented for 1 subject*

All patients had a chest X-ray and the majority (95%) had a high-resolution computed tomography (HRCT) performed (Table 3). The predominant abnormality identified by HRCT at initial evaluation was a ground glass pattern. Only two hospitals performed infant pulmonary function tests. Lung biopsy data were available in 79 of the105 cases reported, with video assisted thoracoscopic biopsy (VATS) predominant. Bronchoalveolar lavage was performed in 65% of patients whilst genetic testing was performed in 25% of cases. Echocardiogram was performed in only 78% of cases

**Table 3:** Investigations performed at time of diagnosis

	*N* (%)	All cases	Diagnosis < 2 years		Diagnosis ≥ 2 years	
		No.reported (Max 107)	*N* (%)	No. reported (Max 66)^a^	*N* (%)	No. reported (Max 40)^a^
*Investigations performed*						
*Chest X-Ray*	99 (100.0)	99	63 (100.0)	63	35 (100.0)	35
Interstitial infiltrates	41 (55.4)	74	27 (54.0)	50	14 (58.3)	24
Alveolar infiltrates	22 (35.5)	62	13 (29.5)	44	9 (50.0)	18
*CT Scan*	95 (95.0)	100	55 (93.2)	59	39 (97.5)	40
*with* controlled ventilation	40 (51.3)	78	30 (69.8)	43	10 (28.6)	35
expiratory views	57 (79.1)	72	32 (76.2)	42	25 (83.3)	30
*CT scan findings*						
Reticular nodular infiltrates	16 (30.7)	52	6 (18.7)	32	10 (50.0)	20
Ground glass pattern	52 (71.2)	73	32 (71.1)	45	20 (71.4)	28
Honey comb pattern	6 (13.1)	46	1 (3.4)	29	5 (29.4)	17
*Lung Biopsy*	79 (75.2)	105	52 (81.3)	64	26 (65.0)	40
*Biopsy Method*						
Open lung biopsy	23 (36.5)	63	18 (43.9)	41	5 (22.7)	22
Video assisted thoracoscopic	40 (63.5)	63	23 (56.1)	41	17 (77.3)	22
Transbronchial	0	63	0	41	0	22
*Echocardiogram*	79 (78.2)	101	47 (77.0)	61	32 (80.0)	40
*Pulmonary HT*	17 (22.1)	77	12 (26.1)	46	5 (16.1)	31
*Pulmonary Function Test*	28 (26.7)	105	2 (3.1)	65	26 (65.0)	40
Infant pulmonary function test	2 (1.9)	102	2 (3.2)	63	-	-
*Sleep Study*	44 (64.7)	68	31 (65.9)	47	1 2 (60.0)	20
*BAL*	54 (65)	83	27 (57.4)	47	26 (74.3)	35
*GORD*	10 (10.2)	98	5 (7.6)	66	5 (15.6)	32
*Genetics Investigations*	25 (25.0)	100	21 (33.3)	63	4 (10.8)	37
*Histological Second Opinion Sought*	30 (49.2)	61	19 (50)	38	11 (47.8)	23

*BAL* Bronchoalveolar lavage; *GORD* Gastro-oesophageal reflux disease; *CT* Computed tomography; HT, Hypertension

^*a*^ *Age at diagnosis was not documented for 1 subject*

In regard to therapy, oxygen was used in the majority of patients (56%) and systemic corticosteroids were the preferred pharmacotherapy initiated on diagnosis (Table 4)

**Table 4:** Treatment of patients at reporting tertiary paediatric centre

	*N* (%)	All cases	Diagnosis < 2 years		Diagnosis ≥ 2 years	
		No. reported (Max 107)	*N* (%)	No.reported (Max 66)^a^	*N* (%)	No. reported (Max 40)^a^
*Treatment given*						
*Steroids*	62 (59.6)	104	33 (52.4)	63	29 (72.5)	40
Response seen	28 (62.2)	45	16 (72.7)	22	12 (52.2)	23
*Hydrochloroquine*	36 (34.6)	104	24 (38.1)	63	12 (30.0)	40
Response seen	12 (63.1)	19	9 (69.2)	13	3 (50.0)	6
*Azithromycin*	26 (25.0)	104	17 (27.0)	63	9 (22.5)	40
Response seen	6 (50.0)	12	2 (40.0)	5	4 (57.1)	7
*Azathioprine*	4 (3.9)	103	1 (1.6)	62	3 (7.5)	40
Response seen	1 (50.0)	2	1 (100.0)	1	0 (0.0)	0
*Cyclophosphamide*	6 (5.8)	104	0 (0.0)	63	6 (15.0)	40
Response seen	1 (33.3)	3	-	0	1 (33.3)	3
*Oxygen*	23 (56.1)	41	17 (56.7)	30	6 (54.5)	11
Response seen	4 (80.0)	5	4 (100.0)	4	0 (0.0)	1

^*a*^ *Age at diagnosis was not documented for 1 subject*

Clinical outcome was defined as good if there was an improvement in respiratory symptoms, return of weight gain and growth towards normal, improvement in exercise tolerance and improvement in lung function and oxygen saturation at rest. At the time of reporting, a good clinical outcome was reported in 79.3% of patients (Table 5). The mortality rate was 6.9% (7 of 101 cases).

**Table 5:** Clinical outcome

	*N* (%)	All cases	Diagnosis < 2 years		Diagnosis ≥ 2 years	
		No. reported (Max 107)	*N* (%)	No reported (Max 66)^a^	*N* (%)	No reported (Max 40)^a^
*Clinical Progress*						
Good	80 (79.3)	101	52 (82.6)	63	27 (73.0)	37
Poor	14 (13.8)	101	6 (9.5)	63	8 (21.6)	37
Died	7 (6.9)	101	5 (7.9)	63	2 (5.4)	37
*Radiological Changes on CT Scan(Post Treatment)* ^*b*^						
Improved	38 (48.7)	78	28 (54.9)	51	9 (34.6)	26
No Change	11 (14.1)	78	4 (7.8)	51	7 (26.9)	26
Deterioration	6 (7.7)	78	2 (3.9)	51	4 (15.4)	26
Not Done	23 (29.5)	78	17 (33.3)	51	6 (23.1)	26

^*a*^ *Age at diagnosis was not documented for 1 subject*

^*b*^ *Radiological changes assessed by the local physician and/or radiologist; no formal grading was undertaken*

## Discussion

This is the first study to describe the diagnoses and characteristics of patients with chILD from Australia and NZ. Over a period of a decade, 115 patients with chILD were identified with a period prevalence of 1.5/million children aged 0–18 years. To date, collaborative efforts estimating frequency of chILD have been limited to Europe and the United States. The incidence of childhood ILD (then termed idiopathic interstitial pneumonitsis) has been reported as 3.6 cases per million in the United Kingdom and Ireland in 2002 [8], similar to a German study which estimated an incidence of 0.13 cases per 100,000 children less than 17 years of age in 2009 [12]. Although we report period prevalence in Australasia, one limitation is that we were not able to calculate incidence, as we did not specifically identify new patients; rather we collected data on patients seen at any time point within the time-frame of the study.

The presenting clinical manifestations of chILD are often subtle and non–specific; they include cough, dyspnoea, tachypnoea, chest wall retractions, exercise limitation and frequent respiratory infections [13]. In term infants, chILD may present as unexplained respiratory failure requiring mechanical ventilation [13, 14]. Unexplained tachypnoea was the most common presenting symptom in our cohort of cases less than 2 years of age, with 38% requiring respiratory support with mechanical ventilation. In older children, exertional dyspnoea was the most common presenting feature demonstrating that chILD should be considered as a differential diagnosis once commoner causes are excluded. Our findings of clinical presentation are similar to those reported in other series [8, 15].

Our study highlights variations between centers in the approach to investigations and management of chILD. For example, echocardiograms were performed in only 78% of cases despite being recommended in all suspected cases of chILD as part of the initial evaluation to exclude pulmonary vascular and structural heart disease which may mimic symptoms of chILD [5, 16]. Our study revealed minimal use of infant lung function tests (2%). Infant lung function testing can be helpful in characterizing disease severity, particularly in diseases such as neuroendocrine hyperplasia of infancy (NEHI) [17], however its availability is limited to only a few sites in Australasia.

HRCT scans help define the extent of the disease, characterise disease involvement and are more sensitive than X-rays in detecting morphological changes related to child [18]. Additionally, HRCT with controlled ventilation improves the scan output and image quality [19], however only half of all our cases had chest imaging with controlled ventilation. The ATS guidelines do not make a specific recommendation on the use of controlled ventilation [5], in contrast to the European guidelines, which support this approach [20]. Clearly, standardised international protocols, similar to those being undertaken in cystic fibrosis, should be established in order to advance this field.

Genetic testing is a non-invasive investigation, helpful in making the diagnosis and estimating recurrence risk for affected families [21]. Genetic studies for disorders of surfactant metabolism were performed in only 25% of patients in this study. This figure is likely to reflect the fact that the Australian reference genetics laboratory (The Children’s Hospital, Westmead) was only established in 2011, towards the end of our study period. Prior to this, genetic testing had been undertaken in a research laboratory at Johns Hopkins University Hospital, USA as part of a research protocol. There is increasing emphasis on genetic studies in the diagnosis of chILD as genetic diagnosis can help avoid lung biopsy [21, 22].

In patients who underwent lung biopsy (75.2%), video-assisted thoracoscopic surgery (VATS) was the preferred modality (66%). VATS has rapidly developed in the last two decades with lower rate of complications and better post-operative clinical course compared to open lung biopsy, and is the recommended approach by the US chILD committee [5, 23]. In contrast, a retrospective review of outcomes in infants with suspected chILD who had all undergone open lung biopsies at a single centre in London, found the procedure to be safe, with few adverse effects directly related to the procedure [24]. The European guidelines do not make recommendations on the type of surgical approach [20]. The benefit of surgical biopsy remains controversial, particularly in well patients and in those with persistent tachypnoea of infancy [25]. However histological diagnosis may guide treatment decisions, especially withdrawal of care [24]. It was reassuring that in our study none of our patients underwent a transbronchial biopsy which is not recommended.

Oxygen (56%), corticosteroids (60%) and hydroxychloroquine (35%) were the main therapeutic modalities of treatment in our cohort. Azithromycin was used in nearly a quarter of cases. The choice and use of these medications was center dependent, reflecting the lack of an evidence base on which to standardize treatment and the lack of parameters on which to define a good clinical response. There are no controlled studies of therapeutic interventions for chILD. In order to partially address this, Bush et al recently published the results of a Delphi consensus process with clinicians from Europe, North America and Australia to harmonise and unify the approach of diagnostic and treatment protocols [20].

Our study identified children with a wide spectrum of diagnostic labels (Table 1) and we also included patients who had not undergone a biopsy. Soares et al [22] retrospectively reviewed 93 chILD cases from Vanterbilt Children’s Hospital between 1994 and 2011; only 68.8% of their cases had a lung biopsy similar to our study (75%). In those who had a biopsy we relied solely on the local histopathology report and the information provided to us by the clinicians which is a limitation of this study as we did not specifically re-examine the biopsy specimens in the context of the chILD classification published in 2007, nearly half way through our study period. As no chILD clinical or research network previously existed in Australasia, it was common practice to refer histological specimens for a second opinion overseas which was done in nearly half (49.2%) of all our cases. In Europe, chILD cases are peer reviewed at the time of diagnosis and are revisited at yearly intervals to evaluate patient outcome and diagnosis [20]. Our study has highlighted this gap and efforts to develop a similar peer review model are currently underway in Australasia.

No cases of hypersensitivity pneumonitis (HP) were identified in our cohort, similar to the UK and Ireland national survey over a 3- year period (1995–1998) [8]. In contrast, 23 cases were identified in Germany over a period of 3 years using the German Surveillance Unit for Rare Paediatric Disorders [26] and 24 cases were identified from a cohort of 185 chILD cases in the European Taskforce survey (1997–2002) [9]. Buchvald et al. estimated a point prevalence of HP in Denmark of 4 per 1,000,000 children [27], but acknowledged that the number appeared high compared to only 100 paediatric cases reported worldwide in 2002 [28]. In contrast, the reported numbers of children with HP from North America are lower. A study of lung biopsies from 101 immunocompetent children (2–18 years) with ILD from 13 centres over a 4- year period identified only 2 cases of HP [29]. Deutsch et al. identified 2 cases from a cohort of 187 under 2 years of age with ILD who underwent a biopsy from 11 centres between 1999 to 2004 [15] similar to Soares et al who identified 2 of 93 patients in a retrospective review over 18 years from a single center [22]. It is possible that some of our cases, such as those reported as usual interstitial pneumonitis, eosinophilia and follicular bronchiolitis, represented undiagnosed hypersensitivity pneumonitis. One potential limitation is that questions pertaining to parental smoking and environmental exposures were not included in the questionnaire although the inclusion of these questions would not have affected the patient diagnoses, as this was a retrospective study. Future prospective studies should include specific questions on environmental exposures.

The classification of chILD has evolved over recent years with changing disease terminologies, improved understanding of genetic causes and identification of new diseases such as NEHI. A recent systematic review undertaken by our group found that four classification systems were published during the time of our study [1] further highlighting the need for future international collaborative attempts to peer review and agree on similar terminology and codes if this field of science is to be advanced further to benefit patients with chILD.

Further limitations of our study are inherent to its retrospective nature and the reliance on recall for identification of cases. The reported numbers likely underestimate the true prevalence in this geographical region; there are no dedicated chILD referral centers in Australasia, and with access to expertise often dispersed geographically, it is likely that cases identified from specific centers represent under-reporting of all true cases which is further compounded by the fact that only 9 out of 12 tertiary centres responded to the survey. Under-reporting is evident from the fact that we identified seven additional cases of chILD that were missed on recall by cross referencing the two genetic laboratories and the variance in the number of diagnosis received from each center. Furthermore, although the death rate in the under 2 year olds (7.9%) was higher than that reported by the European task force cohort (1.7%) [9], it was much lower than the 30.2% reported by Deutsch et al [15]. We tried to minimise recall bias and attempted to improve the accuracy of the recall by the treating physicians with the help of a well-structured, standardised questionnaire based on a questionnaire from a previous prevalence survey from the UK [8]. However, in many cases data on treatment and duration of follow up requested in the questionnaire were not provided. In part, because of these deficiencies in the data, we are unable to draw firm conclusions on the effectiveness of various therapeutic interventions. Despite these limitations, the strength of this study is that we report data on 115 cases of chILD, which we believe significantly adds to the body of literature in these rare childhood disorders.

Similar to reports from other geographical regions in the world, the investigations, diagnosis and management of chILD cases are not standardised, with center variation likely influenced by local biases, resources and expertise. Over the last few years major collaborative efforts in the US and Europe have worked towards achieving consensus protocols and guidelines on the diagnosis and initial treatment of chILD [5, 20]. Much progress has been made in these geographical regions with the development of excellent models for strategic planning for chILD in order to improve the fragmentation of services, enable patients and health professionals to provide and use best practice care. Thus, there is a compelling need for a similar approach in Australasia. We need to establish a local chILD network to build on current expertise in disease diagnosis and treatment and collaborate with international chILD groups. We believe that, in keeping with the principles of a call for a National Plan for Rare Diseases in Australia [6], this study will help raise awareness of the burden of chILD and highlight the need for national and international collaboration to improve the health care for children with interstitial lung disease in Australasia.

## Conclusion

In summary, chILD is rare in Australasia with an estimated period prevalence of 1.5/million children aged 0–18 years. There is a need to establish a geographically local chILD network and work collaboratively with global partners if there are to be major advances in this field of rare childhood lung diseases.

## Abbreviations

ABCA3: ATP-binding cassette sub-family A member 3 protein
chILD: Childhood interstitial lung disease
HP: Hypersensitivity pneumonitis
HRCT: High resolution computed tomography
ILD: Interstitial lung disease
NEHI: Neuroendocrine hyperplasia of infancy
VATS: Video-assisted thoracoscopic surgery
